# Short-Term Renal Replacement Therapy Outcomes of Critically Ill Patients of Acute Kidney Injury and Acute on Chronic Kidney Disease

**DOI:** 10.7759/cureus.78183

**Published:** 2025-01-29

**Authors:** Mahboob Alam, Himansu S Mahapatra, Ranvinder Kaur, Lalit K Pursnani, Muthukumar Balakrishnan, Renju Binoy, Tanvi Thakker, Beauty Suman, Abhishek Jha

**Affiliations:** 1 Department of Nephrology, Atal Bihari Vajpayee Institute of Medical Sciences & Dr. Ram Manohar Lohia Hospital, New Delhi, IND; 2 Department of Critical Care Medicine, Atal Bihari Vajpayee Institute of Medical Sciences & Dr. Ram Manohar Lohia Hospital, New Delhi, IND

**Keywords:** acute kidney injury (aki), continuous renal replacement therapy (crrt), intensive care unit (icu), intermittent hemodialysis (ihd), sustained low-efficiency dialysis (sled)

## Abstract

Background and aims: Sustained low-efficiency dialysis (SLED) is a cost-effective alternative to continuous renal replacement therapy (CRRT) in critically ill acute kidney injury (AKI) patients, in addition to intermittent hemodialysis (IHD) as a mode of renal replacement therapy (RRT) in such patients. This single-center, prospective, observational study aimed to assess the short-term outcomes of SLED and IHD in such patients.

Methodology: Adult (>18 years) patients with AKI requiring dialysis were included from different ICUs of a tertiary care center. Patients were subjected to SLED or IHD according to the standard Kidney Disease: Improving Global Outcomes (KDIGO) 2012 criteria. Treatment duration and ultrafiltration rates were adjusted based on individual patient needs and hemodynamic stability was recorded. Dialysis-free survival, renal function recovery, and mortality rates at one month post discharge were analyzed among all RRT groups.

Results: Out of 128 ICU patients requiring dialysis, 78 underwent SLED, while 43 received IHD. Overall, the mean age was 44.53 years. Patients were predominantly male (53.7%), with common co-morbidities such as hypertension (21.5%) and diabetes mellitus (18.2%). Sepsis (59.2%), hypoperfusion (16.7%), and pregnancy-related AKI (14.16%) were the predominant causes of AKI. Indications for RRT initiation included refractory fluid overload, metabolic acidosis, and refractory hyperkalemia. Patients in the IHD group were relatively younger, had fewer comorbidities, and had more females than those in the SLED group. Thirty-day mortality in the SLED group was significantly higher than that in the IHD group (61.2% versus 20.9%, p < 0.05). Multivariate regression analysis identified vasopressin requirement, mechanical ventilation, and Sequential Organ Failure Assessment (SOFA) scores > 12 as predictors of mortality.

Conclusion: Although IHD is an option of RRT in reasonably stable patients, SLED is also a cost-effective option for hemodynamically unstable AKI patients, particularly in resource-limited settings.

## Introduction

Acute kidney injury (AKI) is a major global public health problem that can occur both in the community and hospital settings. It continues to be one of the major problems in critically ill patients worldwide [1]. AKI leads to a significant burden of chronic kidney disease (CKD) and long-term follow-up studies have shown poor renal outcomes even in patients with complete recovery from AKI [2]. AKI is often encountered as the primary cause of CKD in the developing world, which increases demand for dialysis and transplantation [3]. In intensive care unit (ICU) settings, an estimated 8-12% of patients receive renal replacement therapy (RRT) for severe AKI [4]. Of AKI patients requiring RRT who survive a hospitalization, 5-20% will continue receiving RRT at discharge [5]. The use of RRT among ICU patients is associated with increased length of stay and mortality rates of 50-60% [6,7].

The various modes of RRT that can be offered to these patients are intermittent hemodialysis (IHD), sustained low-efficiency dialysis (SLED), acute peritoneal dialysis (PD), and continuous RRT (CRRT) each with its own pros and cons [8]. No individual modality has been shown to confer mortality benefit over the other. Limited data are available regarding the role of SLED in AKI in the Indian population. In ICU patients with unstable vitals where conventional hemodialysis could not be used as a modality of RRT, CRRT is the preferred modality. However, India being a low and middle-income country with limited resources and healthcare facilities, CRRT is not readily available. SLED is being increasingly adopted as a cost-effective and resource-sparing option. Addressing this gap is essential to provide evidence-based recommendations tailored to the Indian population. Bridging this gap would also contribute to global literature by providing insights into the performance of SLED in diverse healthcare systems and patient populations.

For patients with hemodynamic instability, SLED has become a viable option for CRRT. SLED uses standard hemodialysis machines to deliver prolonged duration RRT (eight to 12 hours compared to three to four hours with classic IHD). While anticoagulation is frequently required for CRRT, SLED can be easily carried out without it [9]. A SLED session can be organized around tests and procedures and is therefore less likely to be interrupted. Comparable clinical results and, in some studies, lower costs and lesser demand on nursing time have been reported by observational studies in patients treated with SLED as opposed to CRRT [10-16].

A randomized trial indicated a similar degree of hemodynamic stability with SLED and CRRT [17]. One small trial found no difference between SLED and continuous venovenous hemofiltration (CVVH) in terms of mortality, but SLED was associated with shorter lengths of stay and duration of mechanical ventilation [9]. In another study by Mishra et al., SLED was shown to have good hemodynamic tolerability in patients with septic shock and AKI [18]. While these studies highlight the clinical potential of SLED as an effective and resource-efficient modality, several limitations exist in the available studies. The majority of data have been generated in non-Indian populations, raising concerns about their applicability to the Indian context, given differences in patient demographics, healthcare systems, and resource availability. Most Indian studies have limited their generalizability by focusing on specific patient populations, such as pediatric patients or sepsis-associated AKI. These limitations highlight the critical need for well-designed, large-scale studies in the Indian population to address these gaps and validate the efficacy and feasibility of SLED in this unique setting.

Thus, the present study was conducted with the aim to describe short-term (30 days) RRT outcomes of AKI and acute on CKD (AOCKD) in critically ill adult patients, with comorbidities and with diverse etiologies of AKI. Additionally, in this study, SLED was also compared with IHD in dialysis requiring AKI patients in the ICU.

## Materials and methods

Study design and duration

This prospective observational study was conducted over a period of November 2022 to May 2024 at a tertiary care teaching hospital in New Delhi, India, after obtaining approval from the Institutional Ethics Committee (IEC) of Atal Bihari Vajpayee Institute of Medical Sciences & Dr. Ram Manohar Lohia Hospital, New Delhi (IEC approval number: ABVIMS/RMLH/1510; dated: 19.01.2024).

Inclusion and exclusion criteria

Patients were enrolled from all ICUs in our hospital, equipped with advanced monitoring systems and ventilators, and staffed by a multidisciplinary team, enabling it to handle complex cases, including trauma, organ failure, and postoperative care. All AKI patients >18 years old, fulfilling any of the criteria for the requirement of dialysis as per the Kidney Disease: Improving Global Outcomes (KDIGO) 2012 guidelines were included and patients with end-stage renal disease (ESRD) were excluded.

Sample size

The sample size was calculated based on the mortality rate. Considering the mortality rate of 59.7% in SLED in AKI with septic shock patients, as reported by Mishra et al. (2016) [18], at 95% confidence level and 20% relative error, the required sample size was 65. Considering a 20% attrition rate to account for any loss to follow-up, the required sample size was 78. However, during the study, all consecutive consenting ICU-admitted patients were enrolled.

Data collection

All the patients admitted to different ICUs were screened for AKI and were assessed for indications of RRT. Those requiring RRT were subjected to IHD, SLED, or CRRT as per their clinical status, like blood pressure and vasopressor requirements. Detailed history, clinical examination, and routine investigations were done to decide indications for dialysis.

SLED was initiated and discontinued by dialysis technicians. The blood flow (Qb) was kept at 100-300 ml/min and the dialysate flow (Qd) at 300-500 ml/min. The dialysate was generated with a bicarbonate proportionating system using tap water treated with a reverse osmosis (RO) system. Dialysate composition was sodium at 140 mEq/L, potassium at 2.0-4.0 mEq/L, bicarbonate at 35 mmol/L, and calcium at 3.5 mEq/L. Dialysate temperature was maintained at 37°C. Unfractionated heparin, 2000 U of heparin, was added to 1000 ml normal saline (NS) priming fluid, followed by injecting 500 U/hr in the arterial line for anticoagulation. If a patient was having a bleeding tendency, then heparin-free dialysis was given. The total period of dialysis per day was approximately six hours. The ultrafiltration rate was decided by clinical examination. IHD was given similarly with Qb 300 and Qd 500-600 mL/min. CRRT was also performed as a standard procedure using replacement fluid.

Blood samples were collected at the beginning and one hour after the end of each dialysis session. Blood urea nitrogen (BUN), creatinine, potassium, pH, and bicarbonate were measured in these blood samples. Arterial blood gas analysis was done before and after the procedures. During the dialysis duration, any fall in blood pressure was noted and adjustments in the inotrope dose were made. If the patient developed intractable hypotension despite the infusion of normal saline and increasing the inotrope dose, the dialysis procedure was terminated.

All patients were followed up for 30 days to assess primary outcomes, i.e., all-cause mortality at 30 days, and secondary outcomes such as dialysis-free survival and renal recovery at the 30th day. Further, the length of ICU stay (hours/days) and hospital stay (days) were also analyzed.

Statistical analysis

Data entry was done in Microsoft Excel (Microsoft Corporation, Redmond, WA), with analysis performed using SPSS version 21.0 (IBM Corp., Armonk, NY). Normality was checked using the Kolmogorov-Smirnov test or the Shapiro-Wilk test for a smaller sample size. Categorical variables were presented as counts and percentages, while quantitative data were reported as mean ± standard deviation (SD) or median (interquartile range, IQR) for discrete variables. Statistical comparisons included paired/unpaired t-tests, Mann-Whitney U tests, and chi-square tests, with Fisher’s exact test for small expected values. Univariate and multivariate logistic regressions assessed risk factors for mortality. Kaplan-Meier survival analysis was used to compare survival between SLED and IHD patients. A p-value of less than 0.05 was considered statistically significant.

## Results

**Figure 1:**
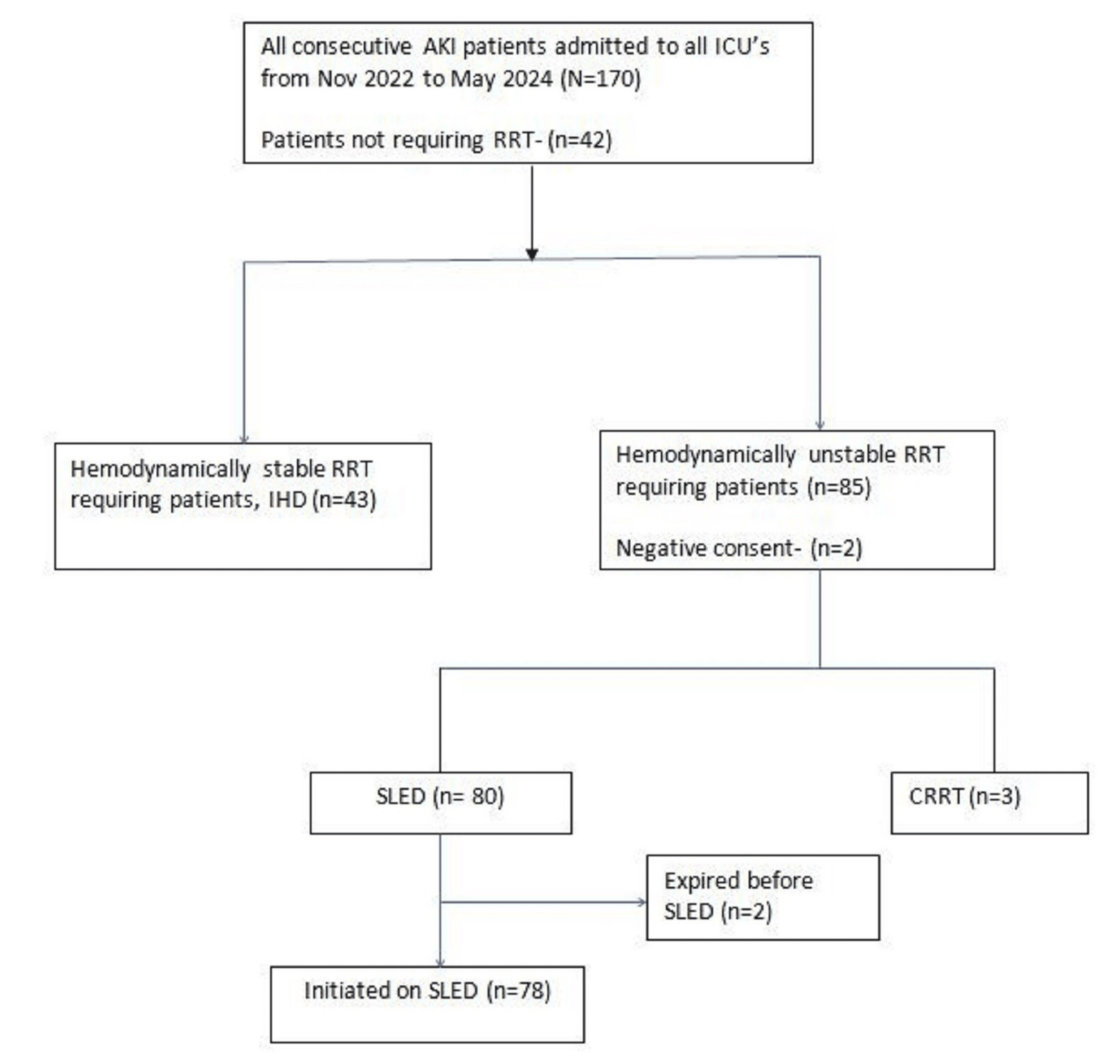
Study algorithm. AKI: acute kidney injury; RRT: renal replacement therapy; ICU: intensive care unit; SLED: sustained low-efficiency dialysis; IHD: intermittent hemodialysis; CRRT: continuous renal replacement therapy.

A total of 170 patients of AKI and AOCKD were admitted to the ICUs. Of those, 42 patients (30.67%) did not require dialysis and 128 patients (69.33%) required dialysis. Of dialysis-requiring patients, 43 were stable and were given IHD. The remaining 85 patients were hemodynamically unstable and required SLED/CRRT. Finally, after excluding patients who underwent CRRT (n = 3), those who gave negative consent (n = 2), and those who expired before RRT initiation (n = 2), 78 patients were given SLED. A total of 216 sessions of SLED and 168 sessions of IHD were conducted during their ICU stay. They were followed up at discharge and one month post discharge to assess outcomes of SLED and IHD in terms of mortality, renal recovery, and dialysis-free survival (Figure 1).

Table 1 outlines the baseline characteristics of the study population. The mean age of the study population was 44.5 years. The age of SLED patients was significantly higher than those in the IHD group (51.21 ± 17.12 years versus 32.60 ± 10.73 years, p = 0.00). The IHD group had significantly more female patients than the SLED group. SLED group had more patients with diabetes mellitus, hypertension, and cardiovascular diseases and more patients on ventilators. Most patients were critically ill and all the patients in the SLED group required inotropic support. A total of 31 patients (39.7%) in the SLED group and six patients (14%) in the IHD group required ventilatory support (p = 0.003). The mean BP of patients in the SLED group was lower than those in the IHD group. Whereas most hematological and biochemical parameters were comparable between SLED and IHD groups, SLED patients had higher total leucocyte count, lower hemoglobin levels, and lower serum creatinine. The most common etiology of AKI among both groups was sepsis, which was distributed equally between the groups. Other common etiologies were ischemic acute tubular necrosis (ATN), and pregnancy-related AKI. Ischemic ATN was significantly more common in the SLED group (23.1% in the SLED group versus 4.6% in the IHD group, p = 0.009) whereas pregnancy-related AKI was a major cause in the IHD group (39.5% in the IHD group versus 2.6% in the SLED group, p = 0.000).

**Table 1:** Comparison of baseline characteristics and lab parameters in SLED and IHD groups. CLD: chronic liver disease; CVA: cerebrovascular accident; BP: blood pressure; SOFA: Sequential Organ Failure Assessment; AKI: acute kidney injury; SLED: sustained low-efficiency dialysis; IHD: intermittent hemodialysis; GN: glomerulonephritis; CA-AKI: contrast-associated AKI; HRS: hepatorenal syndrome; ATN: acute tubular necrosis; Hb: hemoglobin; TLC: total leucocyte count; Na: sodium; K: potassium; SGOT: serum glutamate oxaloacetate transferase; SGPT: serum glutamate pyruvate transferase. Nonparametric data are presented as percentages. Categorical data are compared using the chi-squared test/Fisher’s exact test. Parametric data are presented as mean (SD)/median (IQR) and are compared using the unpaired t-test.

Baseline characteristics	Total (N = 121)	SLED (n = 78)	IHD (n = 43)	P-value
Mean age, years	44.53 ± 15.43	51.21 ± 17.12	32.60 ± 10.73	0.00
Male	65 (53.71)	48 (61.5)	17 (39.5)	0.02
Comorbidities				
Diabetes mellitus	22 (18.2)	20 (25.6)	2 (4.7)	0.003
Hypertension	26 (21.5)	23 (19)	3 (2.5)	0.005
Cardiovascular disease	19 (19)	19 (24.4)	0 (0.0)	0.000
CLD	6 (5.0)	6 (7.7)	0 (0.0)	0.088
Hypothyroidism	2 (100)	2 (100)	0 (0)	0.53
CVA	3 (2.5)	3 (3.8)	0 (0.0)	0.552
Mechanical ventilation	37 (30.57)	31 (39.74)	6 (13.95)	0.003
Mean BP (mmHg)	38.3 ± 16.3	74.6 ± 9.2	99 ± 14.5	0.000
SOFA score	10.55 ± 2.48	11.15 ± 2.65	9.46 ± 1.70	0.000
Etiology of AKI				
ATN	20 (16.5%)	18 (23.1%)	2 (4.6%)	0.009
Sepsis	70 (57.8%)	49 (62.8%)	21 (48.8%)	0.136
HRS	5 (4.1%)	5 (6.4%)	0 (0.0%)	0.09
Obstetric	19 (15.7%)	2 (2.6%)	17 (39.5%)	0.000
CA-AKI	2 (1.6%)	1 (1.3%)	1 (2.3%)	0.667
GN	3 (2.4%)	2 (2.6%)	1 (2.3%)	0.936
Toxic rhabdomyolysis	2 (1.6%)	1 (1.3%)	1 (2.3%)	0.667
Biochemical parameters				
Hb, g/dL	9.33 ± 2.75	8.23 ± 2.29	9.94 ± 2.80	0.001
TLC, n/µL	20136 ± 11293	21810 ± 11898	17101 ± 9496	0.028
Platelets, lakhs/µL	1.85 ± 1.27	1.20 ± 1.06	1.69 ± 1.35	0.460
Serum urea, mg/dL	152.39 ± 67.22	144.36 ± 62.30	165.02 ± 75.91	0.111
Creatinine, mg/dL	6.2 ± 4.01	5.3 ± 3.13	7.9 ± 1.87	0.001
Na, mEq/L	135.41 ± 6.07	136.85 ± 6.07	134.93 ± 7.52	0.289
K, mEq/L	4.68 ± 1.04	4.65 ± 1.00	4.73 ± 1.00	0.684
Total bilirubin, mg/dL	3.33 (0.79-7.15)	1.1 (0.6-1.85)	1.63 (1.15-6.75)	0.058
SGOT, U/L	339 (56-695)	113 (36-467)	142 (82-252)	0.371
SGPT, U/L	215 (40.75-480.75)	82 (34.5-218.5)	84 (46.5-231.5)	0.647
Albumin, g/dL	2.80 ± 0.69	2.8 ± 0.79	2.78 ± 0.48	0.795

Primary outcomes

**Figure 2:**
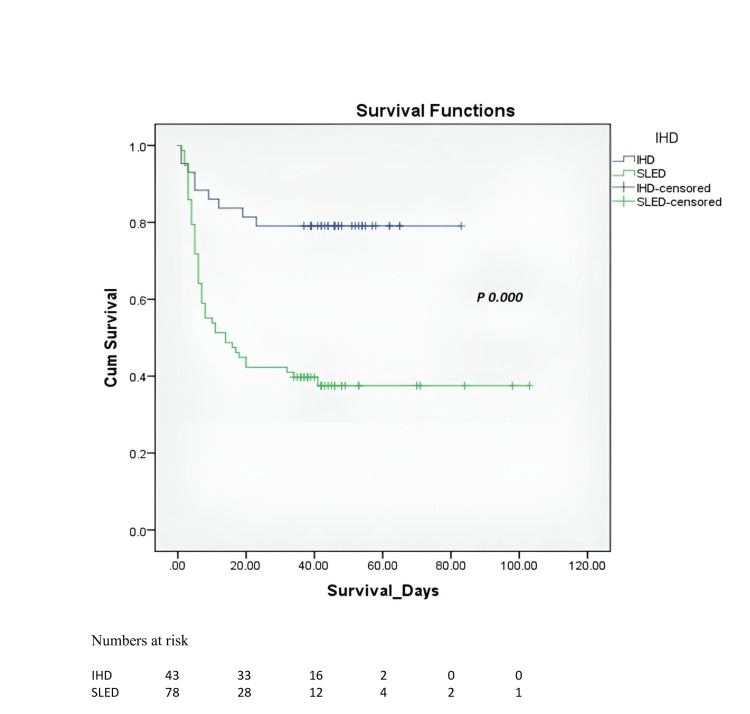
Kaplan-Meier survival curve, SLED versus IHD. SLED: sustained low-efficiency dialysis; IHD: intermittent hemodialysis.

At 30 days, mortality in the SLED group was higher in both intention-to-treat analysis and per-protocol analysis (four of seven (61.5%) versus nine of 43 (20.9%), risk difference = 0.41, 95% CI = 0.243-0.569 and 45 of 75 (59.2%) versus nine of 43 (20.9%), risk difference = 0.38, 95% CI = 0.219-0.547) (p = 0.000; Table 2). Survival analysis was done using Kaplan Meier survival curves, which showed significantly higher survival in the IHD group as compared to that in the SLED group (p = 0.000; Figure 2).

**Table 2:** Results of primary and secondary outcomes at day 30. SLED: sustained low-efficiency dialysis; IHD: intermittent hemodialysis; ITT: intention to treat.

Outcomes		SLED (n = 78)	IHD (n = 43)	Risk difference (SLED-IHD) (95% CI)	P-value
Primary outcomes: 30-day mortality, n (%)	ITT analysis	48 (61.5)	9 (20.9)	0.41 (0.24, 0.57)	0.000
	Per protocol analysis	45 (59.2)	9 (20.9)	0.38 (0.22, 0.55)	0.000
Secondary outcomes	30-day dialysis-free survival	30 (38.4)	22 (51.1)	-0.13 (-0.31, 0.06)	0.177
	30-day kidney recovery	15 (19.2)	7 (16.2)	0.03 (-0.11, 0.17)	0.687
	Duration of ICU admission (days)	12.2 ±13.3	17.4 ± 10.3	-	0.028

Secondary outcomes

There was no significant difference between the SLED and IHD groups in 30-day dialysis-free survival (38.4% versus 51.1%, risk difference = -0.13 (95% CI, -0.311, 0.0573), p = 0.177) and 30-day kidney recovery (19.2% versus 16.2%, risk difference = 0.03 (95% CI, -0.111, 0.17), p = 0.687), as shown in Table 2. In the SLED group, the mean delivered weekly Kt/V urea was 2.13 ± 0.165 with the mean clearance of urea approximately 0.601 per session, whereas in the IHD group, the mean delivered weekly Kt/V urea was 2.92 ± 0.246 with urea reduction ratio around 0.663 per session. Duration of hospital stay was more in the IHD group than in the SLED group (17.4 ± 10.3 days versus 12.2 ± 13.3 days, p = 0.028).

Among SLED patients, the distribution of age, gender, and common comorbidities such as diabetes mellitus and hypertension was similar between survivors and non-survivors, whereas chronic liver disease was significantly higher in non-survivors. Notably, the use of vasopressin and the need for mechanical ventilation were significantly more frequent in non-survivors (p < 0.001). Sequential Organ Failure Assessment (SOFA) scores were also significantly higher in the non-survivor group. Overall, most baseline characteristics were comparable, and key differences in comorbidities and severity indices were observed. ATN was significantly more common among survivors (36.4%) compared to non-survivors (13.3%). Sepsis was prevalent in 68.9% of non-survivors versus 54.5% of survivors, but this difference was not statistically significant (p = 0.19). Hepatorenal syndrome (HRS) was exclusive to non-survivors. Other etiologies such as obstetric causes, contrast-associated AKI, glomerulonephritis, and rhabdomyolysis did not show significant differences between the two groups. The etiology of shock was not different between the two groups.

The efficacy and hemodynamic tolerability of SLED and IHD groups are presented in Table 3. Metabolic acidosis was a common indication for RRT in the SLED group. Whereas in the IHD group, the most common indication for RRT was volume overload. Correction of metabolic acidosis and volume overload was significantly more effective in IHD (p = 0.004 and <0.001) whereas correction of hyperkalemia was comparable between the groups.

**Table 3:** Efficacy and hemodynamic tolerability of SLED between survivor and non-survivor groups. SLED: sustained low-efficiency dialysis; IHD: intermittent hemodialysis. Nonparametric data are presented as percentages. Categorical data are compared using the chi-squared test/Fisher’s exact test. Parametric data are presented as mean (SD)/median (IQR) and are compared using the unpaired t-test.

	SLED		IHD		P-value
	Survivors (n = 33)	Non-survivors (n = 45)	Survivors (n = 34)	Non-survivors (n = 9)	
Indication for SLED					
Acidosis	30 (90.9%)	42 (93.3%)	16 (47.0%)	7 (77.7%)	0.69
Vol. overload	31 (93.9%)	45 (100.0%)	22 (64.7%)	9 (100.0%)	0.17
Hyperkalemia	12 (36.4%)	22 (48.9%)	6 (17.6%)	4 (44.4%)	0.35
Acidosis corrected	30 (100.0%)	14 (31.1%)	16 (100%)	5 (66.7%)	0.004
Volume overload corrected	29 (87.9%)	13 (28.9%)	22 (100%)	7 (77.8%)	0.001
Hyperkalemia corrected	10 (83.4%)	18 (81.9%)	6 (100.0%)	2 (50.0%)	0.8
No. of SLED/IHD done	102	114	128	40	-
Intolerance to SLED/IHD	9 (8.82%)	58 (52.87%)	4 (3.33%)	9 (20.25 %)	<0.0001
Kt/V	0.908 ± 0.121	0.695 ± 0.133	0.983 ± 0.235	0.933 ± 0.256	<0.001

A total of 216 SLED sessions and 168 IHD were performed. However, significantly more SLED patients experienced intolerance to SLED due to a drop in blood pressure and required termination of the session as compared to IHD (p < 0.0001). The dialysis dose delivered, as measured by Kt/V, was significantly lower in the SLED group (0.82 ± 0.236) compared to the IHD group (0.963 ± 0.141, p < 0.001).

Hospital/ICU stay and outcomes

Survivors of SLED had significantly longer ICU and hospital stays compared to non-survivors (p = 0.01 for ICU stay; p < 0.001 for hospital stay).

In-hospital mortality in the SLED group was 57.69%. A total of 33 out of 78 patients were discharged, of whom 2.6% required dialysis. Univariate analysis identified several factors associated with mortality, including vasopressin use, mechanical ventilation, baseline hemoglobin, total bilirubin, alkaline phosphatase, serum albumin, SOFA score > 12, and intolerance to SLED (p < 0.05). In multivariate analysis, the need for vasopressin, mechanical ventilation, and higher SOFA scores were independently associated with mortality (Table 4).

**Table 4:** Multivariate logistic regression analysis for factors associated with mortality. SOFA: Sequential Organ Failure Assessment.

Mortality	Beta coefficient	Standard error	P-value	Odds ratio	Odds ratio, lower bound (95%)	Odds ratio, upper bound (95%)
Vasopressin	2.044	0.833	0.014	7.720	1.509	39.497
Mechanical ventilation	2.405	0.899	0.007	11.079	1.903	64.481
SOFA	0.441	0.164	0.007	1.555	1.127	2.144

These results highlight the critical importance of vasopressor use, mechanical ventilation, and SOFA scores as predictors of mortality.

## Discussion

In recent years, there has been a growing interest in prolonged or extended dialysis modalities, particularly for critically ill patients with AKI in the ICU. SLED offers several advantages, including better hemodynamic stability, flexible treatment schedules, lesser requirement for anticoagulation, and reduced costs. However, studies supporting its outcomes in shock are limited. Our study aimed to describe the above issues during the implementation of SLED in AKI and AOCKD with shock. We will first focus on the outcomes of SLED. Our study aimed to assess the role of RRT in managing ICU patients with AKI and AOCKD, with unstable hemodynamic parameters. All enrolled patients underwent SLED or IHD based on clinical indications. Survivors at discharge were followed up 30 days post discharge to evaluate for 30-day mortality, 30-day renal recovery, and 30-day dialysis-free survival.

In the present study, 30-day mortality was high at 61.5% in the SLED group. This result compares favorably with previous studies. In a prospective study by Mishra et al. (2016), a mortality of 59.7% was reported in 124 patients with septic shock and AKI who underwent SLED [18]. Datt et al. (2017) reported 28 days mortality of 94.2% in the SLED group [19] and Sharma et al. (2020) found a mortality of 61% in AKI patients receiving SLED [20]. In a meta-analysis by Melo et al. (2020), 14 out of 25 studies from developing countries showed mortality of at least 56% (54% average weighted mortality) in RRT requiring hemodynamically unstable patients with AKI [21]. Thirty-day mortality in the IHD group was 20.9%, which was significantly lesser as compared to the SLED group. It can be explained by higher age, more number of comorbidities, and unstable vitals of patients in the SLED group. In a randomized study comparing IHD and CRRT in critically ill patients, a mortality rate of 38% was reported in the IHD arm [22]. So far, no study has directly compared SLED and IHD. However, the PICARD study group conducted a retrospective study where CRRT and IHD were compared and it was concluded that CRRT was associated with a significantly higher risk of mortality and no evidence of a survival benefit was offered by CRRT, though the result could be confounded by the severity of illness [23].

Thirty-day dialysis-free survival (38.4% versus 51.1%, risk difference = -0.13 (95% CI, -0.311, 0.0573), p = 0.177) and 30-day kidney recovery (19.2% versus 16.2%, risk difference = 0.03 (95% CI, -0.111, 0.17), p = 0.687) were comparable between the SLED and IHD groups. These findings highlight that in terms of short-term renal outcomes of AKI, there is no clear advantage of either modality of RRT offered to the patients as suggested in previous studies [24]. This implies that in choosing between SLED and IHD for patients needing dialysis, the decision should be based on the patient's condition, resource constraints, and institutional capabilities.

Dialysis dependence at discharge was 6.4% in the SLED group, which is similar or less as compared to studies from developed countries. Hoste et al. (2008) reported dialysis dependence of 5-20% in survivors of critically ill AKI patients who underwent SLED [5]. Uchino et al. (2005) showed that around 3% of the patients who develop AKI in hospital settings usually end up having long-term dialysis [25].

In multivariate regression analysis, SOFA score > 12, the need for mechanical ventilation, and the need for vasopressin were found to be associated with mortality. In a study by Mishra et al. (2016), SOFA score > 12 was found to be associated with mortality [18]. pH < 7.25 was reported to be associated with mortality by Sharma et al. (2020) [20]. Mishra et al. (2016) reported SOFA score > 12, diabetes mellitus, and vasopressor dependency to be associated with mortality in a cohort of patients with AKI and septic shock [18].

No patients remained dialysis-dependent after 30 days in the SLED group, whereas in the IHD group, dialysis dependence at 30 days was 2.9%. These findings are similar to those reported by Rahhal et al. (2023), who also reported high in-hospital mortality with comparable post-discharge survival and renal recovery outcomes [26]. Another study by Ponce et al. (2013) highlighted similar results, emphasizing mortality risk factors like age and sepsis, while protective factors included urine output and fluid balance [24]. In summary, the current study shows that about one-third of SLED patients achieved 30-day survival without the need for dialysis, highlighting a positive outcome.

By demonstrating SLED as a feasible and cost-effective RRT modality with good renal recovery outcomes in survivors, this study could help shift the focus of ICUs toward implementing and optimizing SLED programs, especially in regions where CRRT is not easily available. This can improve care equity and outcomes for critically ill AKI patients globally.

Our study has certain limitations. It was an observational study. Patients were assigned to the SLED and IHD groups based on their hemodynamic stability. Many of the patients had pre-existing CKD, which is a poor prognostic factor. However, mortality rates in our study remained comparable to previous studies.

## Conclusions

Although mortality was higher in SLED patients, it is a reasonable and cost-effective RRT option for hemodynamically unstable AKI patients who cannot tolerate IHD. SLED has proven to be efficacious, safe, and well-tolerated, particularly at lower doses of vasopressors. This approach is especially crucial in resource-limited settings, where CRRT is often unaffordable and requires additional hardware and nursing care. Unlike CRRT, SLED does not require extra equipment or specialized care and can be administered with the same setup as IHD. Given its feasibility, affordability, and lesser resource requirements, SLED could play a significant role in improving outcomes for critically ill ICU patients in low- and middle-income countries, where CRRT may not be readily available. Expanding the use of SLED could have global relevance, helping to optimize ICU protocols and improve patient care in these settings.

## References

[1] Mehta RL, Cerda J, Burdmann EA (2015). International Society of Nephrology’s 0by25 initiative for acute kidney injury (zero preventable deaths by 2025): a human rights case for nephrology. Lancet.

[2] Gameiro J, Marques F, Lopes JA (2021). Long-term consequences of acute kidney injury: a narrative review. Clin Kidney J.

[3] Cerdá J, Bagga A, Kher V, Chakravarthi RM (2008). The contrasting characteristics of acute kidney injury in developed and developing countries. Nat Clin Pract Nephrol.

[4] Hoste EA, Bagshaw SM, Bellomo R (2015). Epidemiology of acute kidney injury in critically ill patients: the multinational AKI-EPI study. Intensive Care Med.

[5] Hoste EA, Schurgers M (2008). Epidemiology of acute kidney injury: how big is the problem?. Crit Care Med.

[6] Peres LA, Wandeur V, Matsuo T (2015). Predictors of acute kidney injury and mortality in an intensive care unit. J Bras Nefrol.

[7] Luna LD, Soares Dde S, Junior GB (2016). Clinical characteristics, outcomes and risk factors for death among critically ill patients with HIV-related acute kidney injury. Rev Inst Med Trop Sao Paulo.

[8] Jaryal A, Vikrant S (2017). A study of continuous renal replacement therapy and acute peritoneal dialysis in hemodynamic unstable patients. Indian J Crit Care Med.

[9] Schwenger V, Weigand MA, Hoffmann O (2012). Sustained low efficiency dialysis using a single-pass batch system in acute kidney injury - a randomized interventional trial: the renal replacement therapy study in intensive care unit patients. Crit Care.

[10] Kumar VA, Craig M, Depner TA, Yeun JY (2000). Extended daily dialysis: a new approach to renal replacement for acute renal failure in the intensive care unit. Am J Kidney Dis.

[11] Marshall MR, Creamer JM, Foster M (2011). Mortality rate comparison after switching from continuous to prolonged intermittent renal replacement for acute kidney injury in three intensive care units from different countries. Nephrol Dial Transplant.

[12] O'Reilly P, Tolwani A (2005). Renal replacement therapy III: IHD, CRRT, SLED. Crit Care Clin.

[13] Marshall MR, Golper TA, Shaver MJ, Alam MG, Chatoth DK (2001). Sustained low-efficiency dialysis for critically ill patients requiring renal replacement therapy. Kidney Int.

[14] Naka T, Baldwin I, Bellomo R, Fealy N, Wan L (2004). Prolonged daily intermittent renal replacement therapy in ICU patients by ICU nurses and ICU physicians. Int J Artif Organs.

[15] Wu VC, Huang TM, Shiao CC (2013). The hemodynamic effects during sustained low-efficiency dialysis versus continuous veno-venous hemofiltration for uremic patients with brain hemorrhage: a crossover study. J Neurosurg.

[16] Berbece AN, Richardson RM (2006). Sustained low-efficiency dialysis in the ICU: cost, anticoagulation, and solute removal. Kidney Int.

[17] Mishra SB, Azim A, Prasad N, Singh RK, Poddar B, Gurjar M, Baronia AK (2017). A pilot randomized controlled trial of comparison between extended daily hemodialysis and continuous veno-venous hemodialysis in patients of acute kidney injury with septic shock. Indian J Crit Care Med.

[18] Mishra SB, Singh RK, Baronia AK, Poddar B, Azim A, Gurjar M (2016). Sustained low-efficiency dialysis in septic shock: hemodynamic tolerability and efficacy. Indian J Crit Care Med.

[19] Datt B, Ramachandran R, Sharma N (2017). To compare acute peritoneal dialysis with sustained low-efficiency dialysis in critically ill patients requiring renal replacement therapy. Indian J Nephrol.

[20] Sharma R, Maksana M (2020). Safety, efficacy and outcomes of slow low efficiency dialysis (SLED) in critically ill patients with acute kidney injury (AKI) in medical intensive care units (ICU)- a prospective study in a tertiary care hospital in India. Nephrol Dial Transplant.

[21] Melo FA, Macedo E, Fonseca Bezerra AC, Melo WA, Mehta RL, Burdmann EA, Zanetta DM (2020). A systematic review and meta-analysis of acute kidney injury in the intensive care units of developed and developing countries. PLoS One.

[22] Liang KV, Sileanu FE, Clermont G, Murugan R, Pike F, Palevsky PM, Kellum JA (2016). Modality of RRT and recovery of kidney function after AKI in patients surviving to hospital discharge. Clin J Am Soc Nephrol.

[23] Cho KC, Himmelfarb J, Paganini E, Ikizler TA, Soroko SH, Mehta RL, Chertow GM (2006). Survival by dialysis modality in critically ill patients with acute kidney injury. J Am Soc Nephrol.

[24] Ponce D, Abrão JM, Albino BB, Balbi AL (2013). Extended daily dialysis in acute kidney injury patients: metabolic and fluid control and risk factors for death. PLoS One.

[25] Uchino S, Kellum JA, Bellomo R (2005). Acute renal failure in critically ill patients: a multinational, multicenter study. JAMA.

[26] Rahhal A, Najim M, Mahfouz A (2023). Appropriateness and clinical outcomes of short sustained low-efficiency dialysis: a national experience. Open Med (Wars).

